# Effect of chronic administration with human thioredoxin‐1 transplastomic lettuce on diabetic mice

**DOI:** 10.1002/fsn3.2391

**Published:** 2021-06-21

**Authors:** Rie Watanabe, Hiroki Ashida, Mikiko Kobayashi‐Miura, Akiho Yokota, Junji Yodoi

**Affiliations:** ^1^ Laboratory of Infection and Prevention Department of Biological Responses Institute for Virus Research Kyoto University Kyoto Japan; ^2^ Department of Food Science Faculty of Human Life Sciences Mimasaka University Okayama Japan; ^3^ Nanometorics Laboratory Department of Microengineering Graduate School of Engineering Kyoto University Kyoto Japan; ^4^ Graduate School of Biological Sciences Nara Institute of Science and Technology (NAIST) Nara Japan; ^5^ Graduate School of Human Development and Environment Kobe University Kobe City Japan; ^6^ Department of Biochemistry Shimane University Faculty of Medicine Shimane Japan; ^7^ Present address: Institute for Frontier Life and Medical Sciences Kyoto University Kyoto Japan

**Keywords:** Akita mouse, lettuce, nutritional therapy, small intestine, thioredoxin‐1

## Abstract

**Scope:**

Human thioredoxin‐1 (hTrx‐1) is a defensive protein induced by various stresses and exerts antioxidative and anti‐inflammatory effects. Previously, we described a transplastomic lettuce overexpressing hTrx‐1 that exerts a protective effect against oxidative damage in a pancreatic β‐cell line. In this study, we treated diabetic mice (Akita mice) with exogenous hTrx‐1 and evaluated the effects.

**Methods and results:**

Treatment with drinking water and single applications of exogenous hTrx‐1 did not influence the feeding, drinking behavior, body weight, blood glucose, or glycosylated hemoglobin (HbA_1c_) levels in Akita mice. However, chronic administration of a 10% hTrx‐1 lettuce‐containing diet was associated with a significant reduction from the baseline of HbA_1c_ levels compared with mice fed a wild‐type lettuce‐containing diet. It also resulted in an increased number of goblet cells in the small intestine, indicating that mucus was synthesized and secreted.

**Conclusion:**

Our results revealed that the administration of an hTrx‐1 lettuce‐containing diet improves the baseline level of HbA_1c_ in Akita mice. This effect is mediated through goblet cell proliferation and possibly related to protection against postprandial hyperglycemia by mucus, which results in the improvement of blood glucose control. These findings suggest that the hTrx‐1 lettuce may be a useful tool for the continuous antioxidative and antidiabetic efficacies of the hTrx‐1 protein.

## Graphical abstract

Administration of an hTrx‐1 lettuce‐containing diet improves the baseline level of HbA_1c_ in Akita mice. This effect is mediated through goblet cell proliferation in the small intestine and possibly related to protection against postprandial hyperglycemia by mucus, which results in the improvement of blood glucose control. These findings suggest that the hTrx‐1 lettuce may be a useful tool for the continuous antioxidative and antidiabetic efficacies of the hTrx‐1 protein.

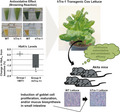

## 1 INTRODUCTION

Oxidative stress caused by excessive amounts of reactive oxygen species (ROS) induces the formation of disulfide bonds in proteins, lipid peroxidation, and DNA cross**‐**linking and causes intractable oxidative stress‐related disorders, such as diabetes mellitus, cancer, and inflammation (Inoguchi et al., 2000; Nishikawa et al., 2000; Sheetz & King, 2002; Tsubouchi et al., 2005). Thioredoxin‐1 (Trx‐1) is a highly conserved 12‐kDa protein with a redox‐active dithiol in its active site (‐Cys‐Gly‐Pro‐Cys‐). It is a defensive molecule induced by various stresses and exhibits antioxidative, antiapoptotic, and anti‐inflammatory effects in all living cells (Watanabe et al., 2010). Trx‐1 is released from cells in response to oxidative stress. Although it has no signaling peptide, it is believed to be involved in signal transduction, proliferation (Yodoi & Tursz, 1991), and extracellular antioxidant processes. However, the extracellular molecular regulation of Trx‐1 remains unclear.

Thioredoxin‐interacting protein (TXNIP)‐related signals regulate glucose metabolism. TXNIP is an important binding partner of Trx and forms a Trx/TXNIP signaling complex (known as redoxisome), which is an evolutionarily conserved reduction–oxidation (redox) signaling complex that plays a significant role in pathophysiology, including diabetes (Yoshihara et al., 2010). TXNIP was shown to bind NOD‐like receptor protein 3 (NLRP3) and activate the NLRP3 inflammasome, which is known to play a significant role in the regulation of the innate immune system (Abderrazak et al., 2015; Ding et al., 2019; Wang et al., 2019). Inflammasome activators, such as ROS, lysosomal instability, and uric acid crystals, promote the dissociation of TXNIP from Trx and lead to an interaction between NLRP3 and TXNIP. The activation of the NLRP3 inflammasome affects glucose tolerance, insulin sensitivity, and interactions with gut microbes (Wang et al., 2019). The inhibition of TXNIP expression, inflammation, and ER stress through the activation of the adenosine monophosphate‐activated protein kinase (AMPK) pathway establishes a protective effect against retinal neurodegeneration in diabetes. This may occur through sulforaphane treatment, which is abundant in broccoli and cabbage and induces the production of Trx protein (Lv et al., 2020; Tanito et al., 2005). Another natural compound, bakuchiol, exhibits multiorgan protective properties owing to its antioxidative and anti‐inflammatory activities. It exerts antidiabetic, antiaging, and anticancer effects by downregulating TXNIP protein levels, upregulating phosphorylation of AMPK, and increasing the levels of Trx‐1 protein and the tight junction protein, occluding, which regulates intestinal mucosal barrier function (Groschwitz & Hogan, 2009; Han et al., 2016; Huang et al., 2015; Liu et al., 2020). Moreover, metformin, which is known as an oral antihyperglycemic agent, has a gastroprotective effect that causes increased mucus production, possibly related to the activation of the AMPK pathway (Noleto et al., 2019). These findings suggest that the activation of the AMPK pathway *via* Trx‐1 regulates increased mucus production and may have a protective effect against diabetes. Recently, it was demonstrated that natural and dietary compounds (e.g., sulforaphane, bakuchiol, and arabinoxylan) exhibit protective effects against diet‐induced metabolic syndrome and diabetes as well as modify the secretion and composition of mucins associated with goblet cells (Li et al., 2019; Tian et al., 2021; Volstatova et al., 2019; Xin et al., 2019).

Overexpression and administration of human Trx‐1(hTxr‐1) protein are effective in a wide variety of animal models for intractable oxidative stress‐related disorders. Trx‐1‐transgenic (Trx‐1 Tg)/C57BL/6 mice, in which hTrx‐1 is systemically overexpressed under the control of the β‐actin promoter, are more resistant to acute pancreatitis (Ohashi et al., 2006) and diabetes (Hamada et al., 2009; Hotta et al., 1998; Yamamoto et al., 2008). In an animal model of type 1 diabetes mellitus (DM1) consisting of nonobese diabetic (NOD) mice, pancreatic β‐cell‐specific overexpression of Trx‐1 significantly reduced the incidence of diabetes (Hotta et al., 1998). In an animal model of type 2 diabetes mellitus (DM2) consisting of obese diabetic db/db mice, β‐cell‐specific Trx‐1 overexpression suppressed progressive β‐cell failure (Yamamoto et al., 2008). These findings suggest that Trx‐1 can protect pancreatic β cells from oxidative stress during both DM1 and DM2.

The administration of exogenous recombinant hTrx‐1 (rhTrx‐1) was evaluated in some animal models, and it was demonstrated that extracellular rhTrx‐1 exerted a protective effect on oxidative stress induced by proinflammatory cytokines, bleomycin, and lipopolysaccharide (Hoshino et al., 2003; Nakamura et al., 2007; Ueda et al., 2006). Continuous infusion of rhTrx‐1 exhibited a significant effect on a bleomycin model, whereas a daily bolus injection or 3‐hr infusion was less effective. These findings suggest that maintenance of a high rhTrx‐1 concentration in the blood is important for its anti‐inflammatory effect.

The mammalian gastrointestinal tract is lined with a single layer of epithelial cells known for their self‐renewal capability. They represent the primary barrier of defense between an organism and its luminal environment (Circu & Aw, 2011; Lipkin, 1973). Control of the redox status at the luminal surface, including the thioredoxin (Trx/TrxSS) redox couple (Watanabe et al., 2010), is important for maintaining mucus fluidity, absorption of nutrients, and protection against chemical‐induced oxidant injury. Goblet cells in the small intestine secrete mucins that form a protective barrier against shear stress and chemical insult. Notch and Wnt/β‐catenin signals control stem cell maintenance and differentiation into the absorptive and secretory cells of the mammalian intestine (Mei et al., 2020). The downregulation of the Notch pathway increases the secretory phenotype of the intestinal cells, which are primarily goblet cells. MAPK suppression in the intestinal crypts increases Wnt/β‐catenin signaling and promotes Paneth and stem cells, whereas the high MAPK signaling activity in the crypts decreases Paneth cells and stem cells and favors goblet cell properties (Heuberger et al., 2014). In addition, cell proliferation and determination of cell fate are controlled by bone morphogenetic protein and PI3K/Akt signaling (He et al., 2004; Scoville et al., 2008). The manner in which these signaling pathways interact to maintain intestinal homeostasis and the effect of dysfunctional signaling on intestinal disorders are not completely understood.

Lettuce (*Lactuca sativa* L.) is an important commercially available crop and an edible leafy species. Therefore, it is a candidate for both the production and delivery of therapeutic proteins. Furthermore, lettuce can be harvested within a few months of sowing and cultivated in indoor hydroculture systems suitable for the growth of genetically modified crops in many countries. Recently, it has been reported that therapeutic proteins and vaccine antigens may be successfully expressed in lettuce (Davoodi‐Semiromi et al., 2010; Ruhlman et al., 2010). We previously created transplastomic lettuce expressing hTrx‐1 (hTrx‐1 lettuce) under the control of the psbA promoter and developed a purification method for hTrx‐1 (Lim et al., 2011). The purified hTrx‐1 (lettuce hTrx‐1) protected MIN6 β‐cells from damage induced by hydrogen peroxide. Moreover, it was shown that lettuce hTrx‐1 exhibited a protective effect against ROS.

C57BL/6‐Ins2^Akita +/−^ diabetic mice (Akita mice) represent a nonobese, hypoinsulinemic animal model of diabetes caused by pancreatic β‐cell failure (Yoshioka et al., 1997). ER stress induced by misfolded proinsulin is responsible for the β‐cell dysfunction and destruction in Akita mice (Cnop et al., 2012; Ozcan & Tabas, 2012). In 10‐week‐old male Akita mice, the blood glucose levels irreversibly increased up to 700 mg/dl, whereas hyperglycemia in female mice was mild. Thus, the Akita mouse is a suitable model for evaluating the effects of hTrx‐1 administration on blood glucose control. In this study, we evaluated the antidiabetic effects following the administration of exogenous hTrx‐1 (microbiologically purified rhTrx‐1 and the lettuce hTrx‐1) to male Akita mice.

## 2 MATERIALS AND METHODS

### 2.1 Mice

Male C57BL/6‐Ins2^Akita +/−^ diabetic mice (Akita mice; 6 weeks old, 15–20 g weight) were purchased from Japan SLC, Inc. Age‐ and sex‐matched mice were utilized for all animal experiments. The mice were maintained in a conventional animal facility and were exposed to a 12‐hr dark/light cycle with food and water ad libitum. All animal experiments were conducted according to the Guidelines for Animal Experimentation at the Kyoto University Graduate School of Medicine and Mimasaka University. All 7‐week‐old Akita mice had high blood glucose levels.

### 2.2 Oral ingestion and intraperitoneal administration of recombinant human Trx‐1 (rhTrx‐1)

Microbiologically purified rhTrx‐1 was provided by Ajinomoto, Inc. For the oral ingestion experiments, rhTrx‐1 water contained 2–5 µg/ml of rhTrx‐1. To evaluate the total amount of food and drink, standard food and H_2_O or rhTrx‐1‐containing water were fed to 9‐week‐old Akita mice for 6 weeks. For body weight monitoring and glycosylated hemoglobin (HbA_1c_) measurements, food and water were provided to 7‐week‐old mice for 18 weeks. Blood samples were collected from the tail vein at the indicated ages. The HbA_1c_ levels were measured as described below.

For the intraperitoneal injection of rhTrx‐1, 7‐week‐old male mice underwent oral glucose tolerance test (OGTT). After overnight fasting (18–19 hr), Akita mice were injected intraperitoneally twice with 40 µg of rhTrx‐1 suspended in 100 µl of phosphate‐buffered saline (PBS; total of 80 µg of rhTrx‐1). D‐glucose (2‐g/kg body weight) was administered orally after 4 hr. Blood samples were taken from the tail vein at the indicated times. The blood glucose levels were measured using the enzyme‐electrode method (glucose PILOT, Aventir Biotech, LLC.).

### 2.3 Preparation and administration of lettuce powder‐containing feeds

Wild‐type (WT) lettuce and green fluorescent protein (GFP)‐ and hTrx‐1‐expressing lettuces were prepared to evaluate the antidiabetic efficacy of hTrx‐1 by oral administration (Lim et al., 2011). The GFP‐expressing lettuce was used as a chronic‐administration control to evaluate the efficacy and safety of the gene transfer technique and its recombinant protein. The leaves of WT and transplastomic lettuces were freeze‐dried by Japan Jiffy Foods, Inc. and powdered (2525BunBun, Niconico Riken). Based on our previously described methods, the concentration of hTrx‐1 protein in dried hTrx‐1‐expressing lettuce was determined to be 6 mg/g of dried leaves. The lettuce powder was admixed with a standard powdered feed (CE‐2, CLEA Japan, Inc.) at a final concentration of 10% (w/w), and all of these foods were blended before being administered to the mice. Akita mice (11–13 weeks old) were fed 10% lettuce powder‐containing diets ad libitum for 13 weeks. Blood samples were taken from the tail vein at the initial and final time points of the lettuce‐containing diet, and the HbA_1c_ levels were measured as described below.

### 2.4 Acute effect of lettuce hTrx‐1 on blood glucose levels in Akita mice

Lettuce powder (100 mg) was suspended in 3 ml of 0.5% methyl cellulose 400 cP solution (w/v), and the powder solution (0.5 g powder/kg body weight) was administered orally to 13‐ to 17‐week‐old Akita mice. Blood samples were collected from the tail vein at the indicated times, and the blood glucose levels were measured using the enzyme‐electrode method.

### 2.5 Protective effect of the lettuce hTrx‐1 on the browning reaction into the lettuce leaves

The powder (15 mg) of WT and the hTrx‐1‐expressing lettuces was resuspended, the supernatants were prepared using 300 µl of RIPA buffer and PBS‐DTT, and the solutions were incubated for 24 hr at 4℃. The RIPA lysis buffer contained 50 mM Tris (pH 7.5), 150 mM NaCl, 10 mM EDTA, 1% Nonidet P‐40, 1% Na‐deoxycholate, 0.1% SDS, and protease inhibitors (Complete, Roche, Schweiz). The PBS‐DTT solution consisted of PBS, 1–5 mM DTT, and protease inhibitors.

### 2.6 Glycosylated hemoglobin (HbA_1c_)

All HbA_1c_ values are expressed as The National Glycohemoglobin Standardization Program values. The HbA_1c_ percentage was determined using DCA 2000 plus (Bayer/Siemens Healthcare Diagnostics) with 1 µl of fresh whole blood.

### 2.7 Histology

The tissue samples were fixed overnight in 4% paraformaldehyde (Wako Pure Chemical Industries). After being embedded in paraffin, the samples were cut into 5‐µm sections. The sections were stained with hematoxylin and eosin (H&E) or Alcian blue (AB)/PAS reagents using standard techniques by the Central Research Laboratory, Okayama University Medical School. The samples were imaged on a BZ‐X700 microscope (KEYENCE).

### 2.8 Goblet cell counting and mucus granule size measurement

The goblet cell counts and mucus granule sizes were evaluated by analyzing goblet cells over a distance on the basement membrane obtained from the stained intestinal sections. All samples were randomized, and all of the positively stained cells from approximately 100 villi were measured in a blinded fashion using a BZ‐X700 microscope and the Adobe Photoshop CC 2017 software.

### 2.9 Transmission electron microscopy (TEM)

Small intestines were fixed with 4% paraformaldehyde/2% glutaraldehyde, followed by 2% osmium tetroxide. The samples were embedded in Quetol‐812, and ultrathin sections were imaged for goblet cells using a JEM‐1200EX electron microscope manufactured by JEOL (Tokai Electron Microscopy, Inc.).

### 2.10 Statistical method

Values are expressed as mean ± SE. Statistical comparisons were performed using Student's *t* test. A statistically significant difference was defined as *p < .05 and **p < .01.

## 3 RESULTS

### 3.1 Administration of recombinant human Trx‐1 (rhTrx‐1) to Akita mice

**FIGURE 1:**
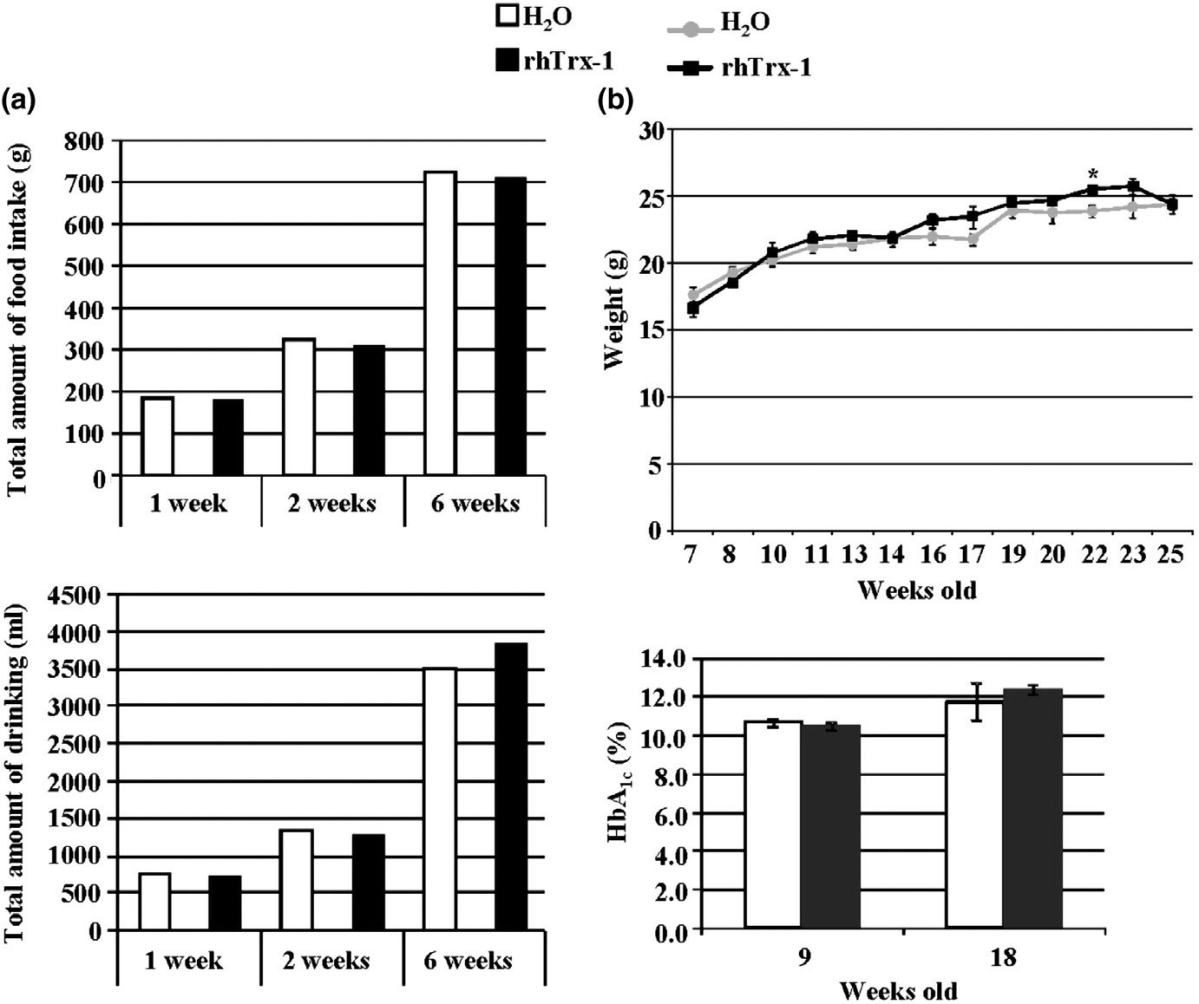
Effect of recombinant hTrx‐1 (rhTrx‐1) on the feeding, drinking, and body weight of diabetic mice. (a) rhTrx‐1 did not influence the total amount of food intake and water consumption. Open column: H_2_O; closed column: rhTrx‐1 water. The rhTrx‐1 water contained 5 µg/ml of rhTrx‐1. Akita mice consumed H_2_O or rhTrx‐1 water with standard feed for 1, 2, and 6 weeks. The total amount of food intake (upper) and water (lower) in five male Akita mice are presented. Nine‐week‐old male Akita mice were utilized. (b) The body weight and HbA_1c_ levels did not change between the H_2_O and rhTrx‐1 water consumption. Upper: H_2_O (circle) and rhTrx‐1 water (square); lower: H_2_O (open column) and rhTrx‐1 water (closed column). The rhTrx‐1 water contained 2–5 µg/ml of rhTrx‐1 in the water. *n* = 5 male Akita mice for H_2_O or the rhTrx‐1 water. *p = .04

To evaluate the effect of oral administration of rhTrx‐1 on diabetic mice, male Akita mice were given water containing 2 to 5 µg/ml of rhTrx‐1 for various times (Figure 1). Standard food and H_2_O or rhTrx‐1 water were provided to five Akita mice ad libitum. The total amount of food and water intake for both groups was calculated at three time points (1, 2, and 6 weeks), whereas feeding and drinking were not influenced by the rhTrx‐1‐containing water in any of them (Figure 1a). Akita mice ingested approximately 3.4–5.0 g of standard food per day in both groups and drank approximately 17–21 ml of H_2_O or rhTrx‐1‐containing water per day. With respect to body weight and glycosylated hemoglobin (HbA_1c_) levels, no significant difference was observed between H_2_O and the rhTrx‐1 water groups (*p* > .05) for the 7‐ to 25‐week‐old animals (Figure 1b). Akita mice weighed approximately 17–26 g (7–25 weeks old) in both groups, and the HbA_1c_ levels were 10.7% ± 0.2% (H_2_O) and 10.5% ± 0.2% (rhTrx‐1 water) at 9 weeks old and 11.8% ± 0.9% (H_2_O) and 12.4% ± 0.3% (rhTrx‐1 water) at 18 weeks old. The HbA_1c_ level of 22‐week‐old mice was 12.9% ± 0.2% in the rhTrx‐1 water group (data not shown). Taken together, long‐term oral administration of rhTrx‐1 through drinking water did not affect the feeding, drinking behavior, body weight, or blood glucose control in Akita mice.

### 3.2 Acute effect of intraperitoneal rhTrx‐1 injection on Akita mice

**FIGURE 2:**
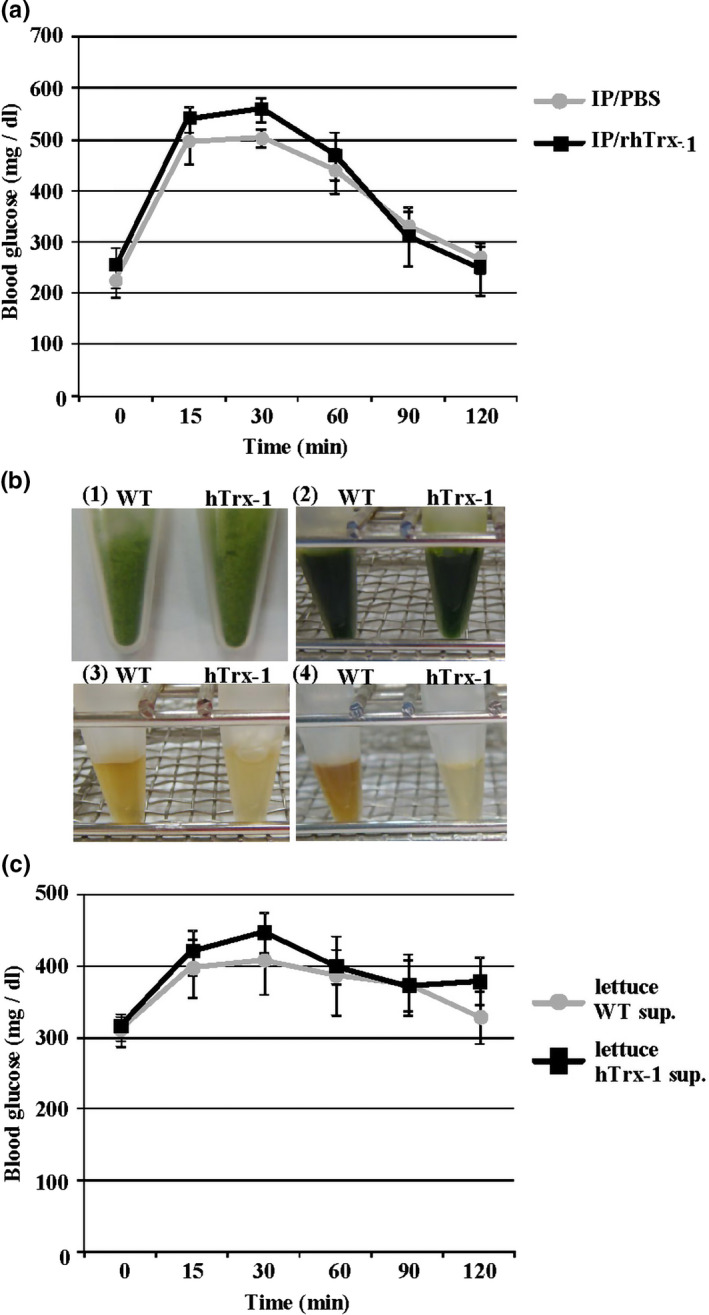
Acute effects of purified hTrx‐1 and lettuce‐containing hTrx‐1 on the blood glucose levels in diabetic mice. (a) Acute effect of an intraperitoneal rhTrx‐1 injection in Akita mice. Mice were injected intraperitoneally two times with PBS or 40 µg of rhTrx‐1 and underwent OGTT for 4 hr. Circle: PBS; square: rhTrx‐1. *n* = 5 male Akita mice for each group. (b) The inhibitory effect of lettuce hTrx‐1 on the browning reaction of the lettuce leaves. (1) WT and the hTrx‐1‐expressing lettuce powders, (2) RIPA, and (3) PBS‐DTT buffers. The powdered lettuces were suspended, the supernatants were prepared, and the solutions were incubated for 5 min at 4℃. (4) The powdered WT and hTrx‐1 lettuces were suspended in PBS‐DTT buffer, and the supernatants were incubated for 24 hr at 4℃. (c) Acute effect of lettuce hTrx‐1 on the blood glucose levels in diabetic mice. Circle: WT lettuce; square: hTrx‐1‐expressing lettuce. *n* = 5 male Akita mice for each group

We investigated an acute effect of intraperitoneal rhTrx‐1 injection on male Akita mice by administering rhTrx‐1 (40 µg, two times) intraperitoneally to the male mice, followed by an OGTT after 4 hr. The results indicated that the blood glucose levels were not different between the PBS and rhTrx‐1 groups at all time points (*p* > .05; Figure 2a), indicating that single‐shot intraperitoneal administration of microbiologically purified rhTrx‐1 did not influence the blood glucose levels of Akita mice.

### 3.3 Protective effect of lettuce hTrx‐1 on oxidative reactions in lettuce leaves and acute administration of lettuce hTrx‐1 to Akita mice

First, we confirmed a protective effect of lettuce hTrx‐1 on an oxidative reaction in lettuce leaves using a simple method, that is, the browning reaction of lettuce leaves induced by oxidation in the air. Figure 2b demonstrates that the hTrx‐1 protein in powdered lettuce leaves inhibited the browning reaction caused by the addition of RIPA and PBS‐DTT buffers. The insulin‐reducing activity of lettuce hTrx‐1 was also demonstrated previously (Lim et al., 2011). These results indicate that the efficacy of the hTrx‐1 protein in powdered lettuce leaves is retained under in vivo conditions, and hTrx‐1 lettuce powder can be administered to Akita mice.

To demonstrate an acute effect of lettuce hTrx‐1 on the blood glucose levels in mice, lettuce powder (0.5 g/kg body weight) was suspended in methyl cellulose and orally administered to male Akita mice. The blood glucose levels were measured at the indicated times using an enzyme‐electrode method (Figure 2c). Although the blood glucose levels increased at the 15‐ and 30‐min time points, no disparity was observed between the WT and hTrx‐1‐expressing lettuces (*p* > .05). Thus, an acute administration of hTrx‐1 did not affect the blood glucose control in Akita mice.

### 3.4 Chronic administration of a 10% lettuce powder‐containing diet to Akita mice

**FIGURE 3:**
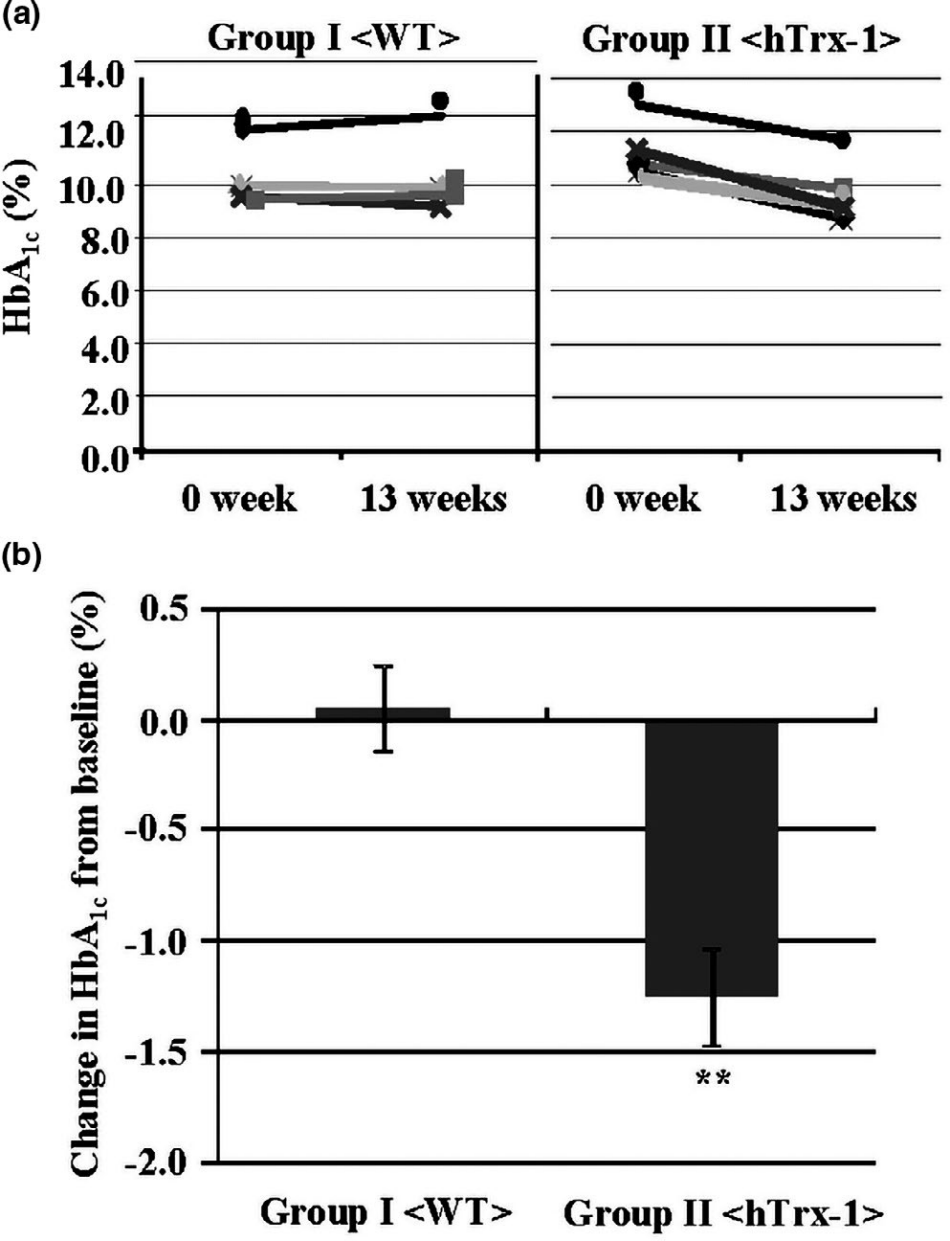
Effect of the chronic administration of a 10% hTrx‐1 lettuce powder‐containing diet on the HbA_1c_ levels in Akita mice. (a) HbA_1c_ levels in the WT and hTrx‐1 lettuce groups. Groups I and II indicate groups administered the WT and hTrx‐1 lettuce‐containing diets, respectively. The HbA_1c_ levels were measured at the initial (0 week: baseline) and final (13 weeks) time points of the lettuce‐containing diets. The graph indicates the raw data for the HbA_1c_ levels. *n* = 4 male Akita mice for group I and 5 male Akita mice for group II. (b) Changes in the HbA_1c_ levels from baselines in these groups. Groups I and II were administered the WT and hTrx‐1 lettuce‐containing diets, respectively. *n* = 4 male Akita mice for group I and 5 male Akita mice for group II. **p < .01, *p < .05

To analyze the effects of a chronic administration of hTrx‐1, a 10% lettuce powder‐containing diet was provided to male Akita mice. After 13 weeks, food intake, body weight, blood glucose, fasting serum glucose, and HbA_1c_ levels were compared between the WT and hTrx‐1 lettuce diet groups (Table 1), and no difference was observed. However, the 10% hTrx‐1 lettuce diet was associated with a significant reduction in the HbA_1c_ levels compared with the WT group (0.1% ± 0.2% in WT and −1.2% ± 0.2% in hTrx‐1; *p* = .003; Table 2; Figure 3). The 10% GFP lettuce diet was also used as a chronic‐administration control for the gene transfer technique and its recombinant protein. The administration of GFP lettuce did not significantly impact recombinant protein excretion, lifetime, food intake, body weight, glucose levels, or a reduction of HbA_1c_ levels compared with the WT group (HbA1c level −0.5% ± 0.1%; *p* = .06). This indicates that our approach with the chronic administration of the recombinant is effective for evaluating hTrx‐1‐related responses (data not shown). These results reveal that the chronic administration of a 10% hTrx‐1 lettuce powder‐containing diet improved blood glucose control in Akita mice.

**TABLE 1:** Food intake, body weight, blood glucose, fasting serum glucose, and HbA_1c_ levels in Akita mice following the administration of a lettuce‐containing diet

	Group I <WT>	Group II <hTrx−1>
	Baseline/0 week	Baseline/0 week
Weight (g)	21.1 ± 0.2	22.7 ± 0.4
Blood glucose (mg/dl)	>600	>600
Fasting plasma glucose (mg/dl)	286.3 ± 34.7	334.0 ± 20.8
HbA_1c_ (%)	10.3 ± 0.6	10.9 ± 0.5
	13 weeks	13 weeks
Food intake (g)	465.3 ± 24.4	469.6 ± 21.3
Weight (g)	23.6 ± 0.5	22.9 ± 0.5
Blood glucose (mg/dl)	>600	>600
Fasting plasma glucose (mg/dl)	320.3 ± 73.1	314.0 ± 80.4
HbA_1c_ (%)	10.4 ± 0.8	9.7 ± 0.5

Note

Groups I and II were administered the WT and hTrx‐1 lettuce‐containing diets, respectively. For the measurement of fasting plasma glucose, the mice were in a fasting state for 20–24 hr. *n* = 4 male Akita mice for group I and 5 male Akita mice for group II.

**TABLE 2:** Changes in the body weight, fasting serum glucose, and HbA_1c_ levels in groups I and II

	Group I <WT>	Group II <hTrx−1>	*p* value
Weight (g)	2.3 ± 0.5	0.8 ± 0.7	.13
Fasting plasma glucose (mg/dl)	34 ± 107.3	−20 ± 32.0	.67
HbA_1c_ (%)	0.1 ± 0.2	−1.2 ± 0.2	.003

Note

Groups I and II were administered WT and hTrx‐1 lettuce‐containing diets, respectively. *p* values: groups I versus II. *n* = 4 male Akita mice for group I and 5 male Akita mice for group II.

### 3.5 The number and area size of goblet cells in the intestinal villi are elevated by feeding an hTrx‐1 lettuce diet to Akita mice

**FIGURE 4:**
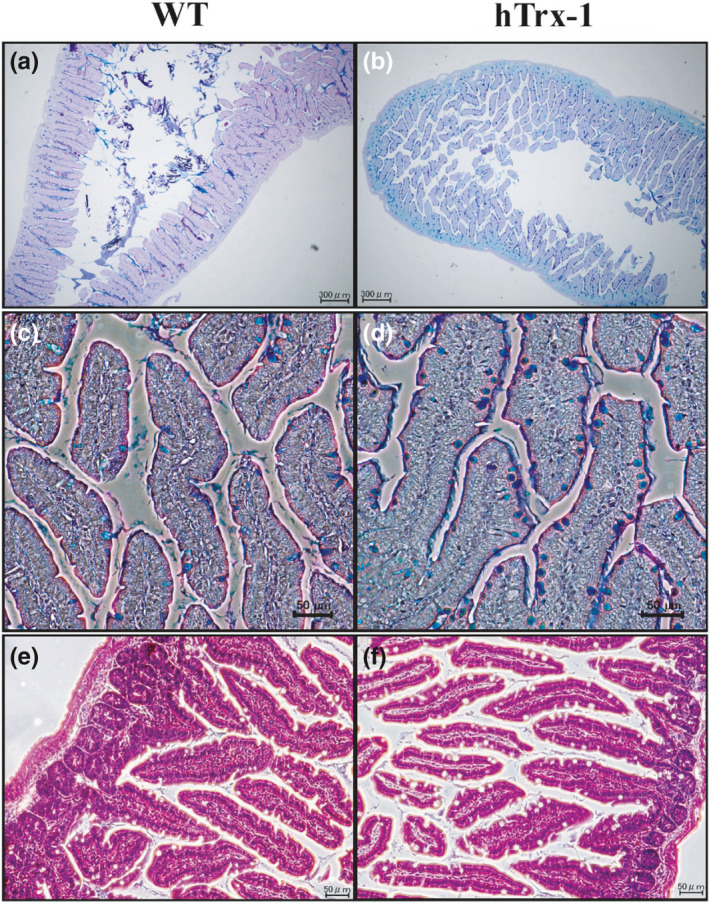
The number of goblet cells in the small intestine increased after the administration of an hTrx‐1 lettuce powder‐containing diet. (a, c, e) WT and (b, d, f) hTrx‐1 lettuce‐containing diets were fed to Akita mice, respectively. (a–d) AB/PAS‐stained small intestinal sections of Akita mice in WT and hTrx‐1 lettuce powder‐containing diets showing goblet cells. (a, b) Magnification = 4×. (c–f) Magnification = 20×. (e, f) Representative H&E‐stained images taken from small intestinal sections of Akita mice. *n* = 4 male Akita mice for the WT group and 5 male Akita mice for the hTrx‐1 group

**FIGURE 5:**
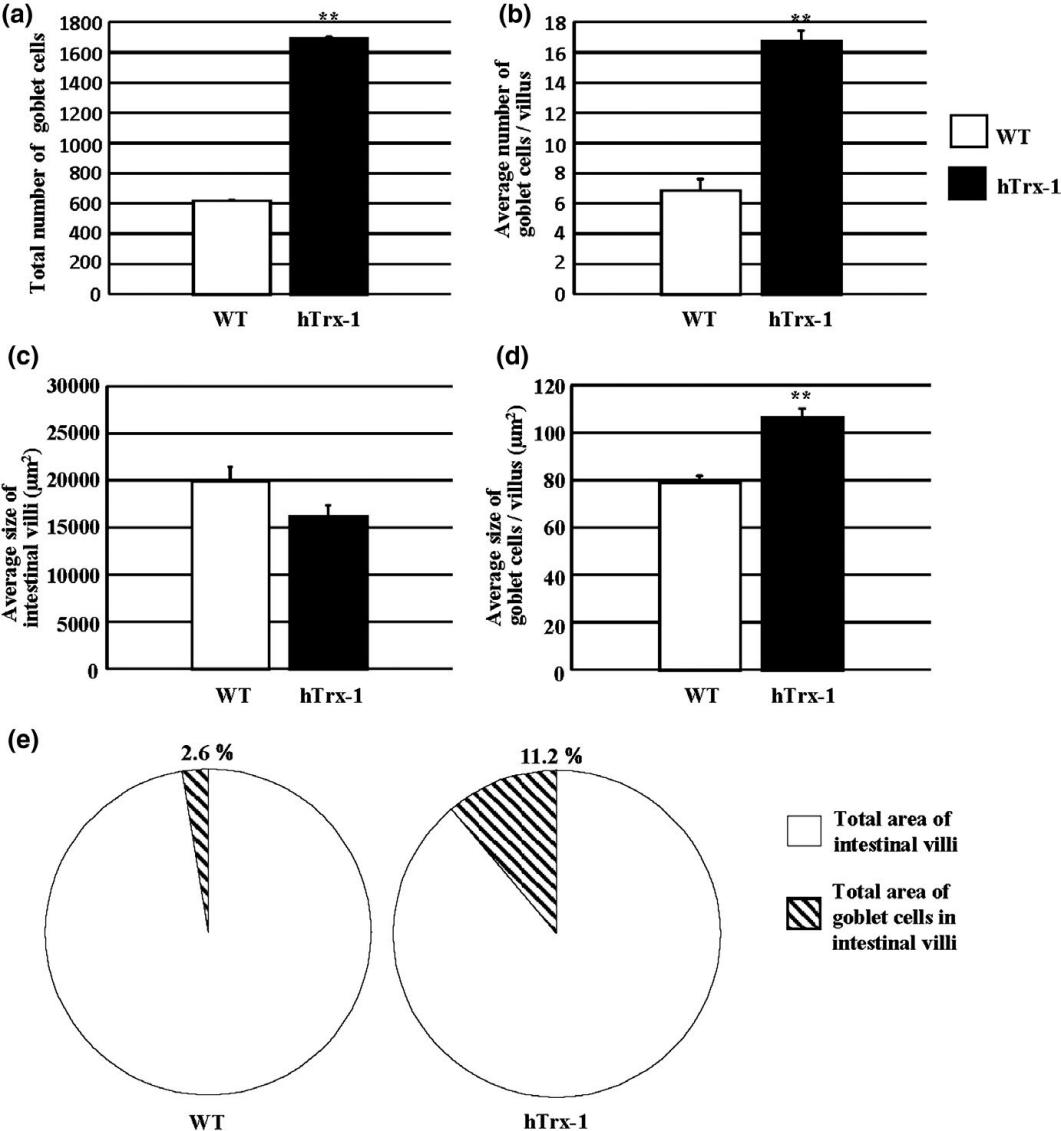
The number and size of goblet cells in the intestinal villi are elevated by feeding Akita mice with an hTrx‐1 lettuce diet. (a–d) Open column: WT and closed column: hTrx‐1 lettuce diets. *n* = 101 villi for the WT and 102 villi for the hTrx‐1 lettuce diet in the small intestines, respectively. (a) Total number of goblet cells in the villi. (b) The number of goblet cells in the villus between the small intestines. (c) The average area of the villi. (d) The goblet cell area in the villus between the small intestines. (e) The area proportion of the goblet cells between the intestinal villi

**FIGURE 6:**
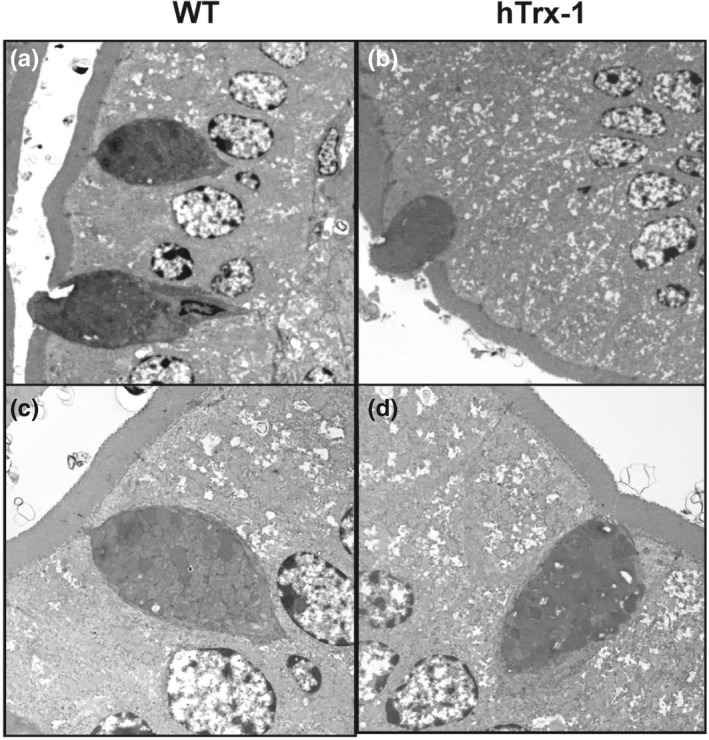
Transmission electron microscopic images of the small intestine between the WT lettuce‐ and hTrx‐1 lettuce diet‐fed Akita mice showing similar morphologies of mucus granules. (a, c) WT and (b, d) hTrx‐1 lettuce‐containing diets, respectively. Magnification = (a, b) 579×, (c, d) 3,550×

The blood levels of recombinant hTrx‐1 between the WT lettuce‐ and hTrx‐1 lettuce‐fed Akita mice were measured using a sandwich enzyme‐linked immunosorbent assay (ELISA) as described previously (Nakamura et al., 1996). The hTrx‐1 protein was nearly undetectable, suggesting that hTrx‐1 in the digested lettuce was not directly translocated into the blood serum for blood glucose control (data not shown). Next, we investigated the histology of the small intestine in the Akita mice (Figure 4). AB/PAS staining surprisingly demonstrated an elevation of the number and area size of goblet cells in the villi by the hTrx‐1 lettuce diet. Figure 5 demonstrates that the number and area size of the goblet cells were significantly augmented in the hTrx‐1‐treated animals. To demonstrate an effect of the hTrx‐1 diet on mucus secretion in the goblet cells, transmission electron microscopy was performed. For both hTrx‐1 and WT diets, the mucus was secreted from the vesicles of the goblet cells in the small intestines in a similar manner (Figure 6), suggesting that mucus secretion was not influenced by the hTrx‐1 diet. Taken together, those results suggest that the dietary intake of hTrx‐1 may be related to an induction of goblet cell proliferation, maturation, and/or mucus biosynthesis in the small intestine but not the manner of mucus secretion.

## 4 DISCUSSION

The administration of exogenous rhTxr‐1 protein has been demonstrated to be effective in animal models for intractable oxidative stress‐related disorders (Hoshino et al., 2003; Nakamura et al., 2007; Ueda et al., 2006). This suggests that maintenance of a high hTrx‐1 concentration in the blood is important for its protective effect on oxidative stress. However, continuous intravenous infusion is associated with pain and limitations to the quality of life. With respect to the oral administration of Trx‐1, it had been demonstrated that indomethacin‐induced gastric injury was reduced by daily food intake of sake yeast‐derived Trx‐1 molecule for 3 days (Nakajima et al., 2012). In addition, it was demonstrated that sulforaphane, which induces Trx protein, has protective effects on diet‐induced metabolic syndrome and diabetes (Lv et al., 2020; Tanito et al., 2005; Tian et al., 2021), whereas the effects of exogenous hTrx‐1 on genetically severe diabetes are unknown. In the present study, we administered exogenous hTrx‐1 to Akita mice and evaluated changes in the diet behavior, body weight, and blood glucose control. The administration of rhTrx‐1 and lettuce hTrx‐1 in drinking water as well as single applications did not influence the dietary behavior, body weight, blood glucose, and HbA_1c_ levels in Akita mice (Figures 1 and 2). However, chronic administration of a 10% hTrx‐1 lettuce‐containing diet was associated with a significant improvement from the baseline of HbA_1c_ levels (Figure 3). As a control for the transplastomic lettuce, we treated mice with a 10% GFP lettuce powder‐containing diet, which exhibited no significant difference compared with the WT group (data not shown). However, the 10% hTrx‐1 lettuce‐containing diet resulted in a significant reduction in HbA_1c_ levels compared with the WT diet. These results indicated that the blood glucose control in Akita mice was improved by the daily oral administration of hTrx‐1‐containing lettuce. In addition, this approach was not associated with pain or any limitations to the quality of life. Furthermore, Figure 2b demonstrates that hTrx‐1 lettuce exhibited a protective effect on the browning reaction of lettuce leaves, indicating the added value of hTrx‐1 transplastomic lettuce.

We performed an hTrx‐1 ELISA in blood plasma after hTrx‐1 administration (data not shown). In plasma from the lettuce‐fed Akita mice, hTrx‐1 was undetectable by this assay. Moreover, the fasting serum glucose levels were not affected by the WT or hTrx‐1 lettuce‐containing feed, whereas the hTrx‐1 lettuce diet was associated with a reduction in the HbA_1c_ levels (Figure 3). These findings suggest that orally administered hTrx‐1 protein is protected from gastric digestion by the plant cell wall and may act on small intestinal epithelial cells, but not in the blood. Furthermore, Figure 5 demonstrates that the number and area size of goblet cells, which secrete mucins to form a protective barrier against shear stress and chemical insult, were elevated in the intestinal villi of the hTrx‐1 lettuce‐fed group. Collectively, our observations suggest that the hTrx‐1 molecule in the lettuce diet may act in the small intestine to decrease postprandial glucose levels by promoting goblet cell proliferation, maturation, and/or mucus biosynthesis but not the manner of mucus secretion (Figure 6; Wlodarska et al., 2014). Contrarily, previous studies demonstrated that dietary compounds, which interact with goblet cells, can modify the secretion and composition of mucins (Volstatova et al., 2019).

TXNIP, a Trx‐binding partner, activates the NLRP3 inflammasome with activators, such as ROS production, lysosomal instability, and uric acid crystals. The activation of the NLRP3 inflammasome affects glucose tolerance, insulin sensitivity, and interactions with gut microbes (Ferreira et al., 2019; Yoshihara et al., 2010). NLRP6 is also a component of a cytoplasmic inflammasome protein complex that promotes innate immune response. NLRP6 inflammasome signaling in the intestinal goblet cells stimulates autophagy required for mucus production and resistance to infection (Ghimire et al., 2020; Wlodarska et al., 2014; Yin et al., 2019). Trx‐1 may participate in NLRP6‐mediated signaling. The inhibition of the TXNIP expression through the activation of the AMPK pathway induces protective effects against diabetes following treatment with bakuchiol or sulforaphane, which induces the Trx protein (Groschwitz & Hogan, 2009; Han et al., 2016; Huang et al., 2015; Liu et al., 2020; Lv et al., 2020; Tanito et al., 2005). In addition, metformin, an oral antihyperglycemic agent, exerts a gastroprotective effect through increased mucus production, which is possibly related to the activation of the AMPK pathway (Noleto et al., 2019). These findings suggest that during the activation of the AMPK pathway *via* the Trx‐1 functional regulation, increased mucus production may exert a protective effect against diabetic conditions. In addition to sulforaphane, numerous functional compounds contained in natural foods have been shown to be related to the NLRP3 inflammasome‐mediated IL‐1β regulation and redox reactions (Tozser & Benko, 2016). This suggests that Trx‐1, as the redoxisome, may participate in the NLRP3 and NLRP6 inflammasome as a component of the complex.

The HbA_1c_ levels at the end point remained high in group II (Table 1), even though the administration of hTrx‐1 lettuce improved the blood glucose control in Akita mice. This suggests that hTrx‐1 lettuce may be desirable for use in combination with other hypoglycemic agents, such as insulin analogs, GLP‐1 analogs, or DPP‐4 inhibitors.

In summary, our results revealed that dietary administration of hTrx‐1 lettuce improved the baseline level of HbA1c in Akita mice, which was mediated through goblet cell proliferation, maturation, and/or mucus biosynthesis in the small intestine. The effect may be related to protection against postprandial hyperglycemia with mucus and results in the improvement of blood glucose control. These findings suggest that the hTrx‐1 lettuce may be a useful tool for the continuous antioxidative and antidiabetic efficacy imparted by the hTrx‐1 protein and may contribute to nutritional therapy for patients with diabetes.

## CONFLICTS OF INTEREST

The authors have declared no conflicts of interest.

## AUTHOR CONTRIBUTIONS

**Rie Watanabe:** Conceptualization (lead); Data curation (lead); Formal analysis (lead); Funding acquisition (equal); Investigation (lead); Methodology (lead); Project administration (lead); Resources (equal); Software (lead); Supervision (equal); Validation (equal); Visualization (lead); Writing‐original draft (lead); Writing‐review & editing (equal). **Hiroki Ashida:** Conceptualization (supporting); Methodology (supporting); Resources (supporting); Validation (supporting); Writing‐review & editing (supporting). **Mikiko Kobayashi Miura:** Data curation (supporting); Formal analysis (supporting). **Akiho Yokota:** Conceptualization (supporting); Funding acquisition (equal); Methodology (supporting); Project administration (supporting); Resources (equal); Supervision (equal); Validation (equal); Writing‐review & editing (supporting). **Junji Yodoi:** Conceptualization (supporting); Funding acquisition (equal); Methodology (supporting); Project administration (supporting); Resources (equal); Supervision (equal); Validation (equal); Writing‐review & editing (equal).

## ETHICAL APPROVAL

This animal study was approved by the animal care and use committees, Kyoto University and Mimasaka University (29/30‐01(A)/02, 2019/2020‐01/02).

## Data Availability

The data that support the findings of this study are openly available in MIMARIPO in Mimasaka University Library at https://mimasaka.repo.nii.ac.jp/, reference number: 20210607001 (https://mimasaka.repo.nii.ac.jp/?action=pages_view_main&active_action=repository_view_main_item_detail&item_id=837&item_no=1&page_id=13&block_id=21) and in NPO Japan Biostress Research Promotion Alliance (http://www.jbpa.me/).
